# Vindiciæ Medicæ; or, a Defence of the College of Physicians

**Published:** 1834-07-01

**Authors:** 


*Vindiciæ Medicæ; or, a Defence of the College of Physicians.* London, 1834. 8vo. pp. 113.

A PETITION was presented last year to both houses of parliament, signed by forty-nine licentiates, who complained that they were illegally excluded from the fellowships of the College of Physicians. Now, this word *illegally* was an extravagant flourish, and must have appeared so to every one who knew that the repeated decisions of the Court of King's Bench had always been in favour of the fellows, and against the licentiates, and that the charter and byelaws were consequently sound in law. Sir George Tuthill, however, the author of the pamphlet before us, has taken the trouble to go through the allegations of the petition, and confute them one by one. This was perhaps a work of supererogation, as the flourish is allowed on all hands to have been a flourish; yet it is not to be regretted, as it has made the charter, and the construction put upon it by the judges, more easy of access to many readers.

A far more difficult question remains, not as to what is, but what ought to be the law? We confess that we are not among those who would cite the existence of an institution in the days of Henry the Eighth as a sufficient authority for its existence now; and therefore, if the College of Physicians had nothing but acts of parliament on its side, our advice would be simply to repeal the Acts. It is our opinion, however, that the College is upon the whole a well-regulated and praiseworthy body. It does not persecute—that is, prosecute, (for, in the case of medical corporations, we hold these to be synonymous terms,) and of late years it has shown a reasonable facility in elevating distinguished licentiates to the rank of fellow. We should have no objection to see this facility slightly increased, and we have little doubt that it will be so : indeed, it seems to us much more likely that errors will be committed in granting than in refusing; and it would appear, from the following passage, that the College is about to concede more than any moderate licentiate could require. Surely the titular *alumni* of Aberdeen and Erlangen do not insist on the exclusion of the genuine graduates of real universities?

"The College of Physicians pretends to no exemption from the imperfections of civil institutions; but is fully sensible that as all laws and ordinances ought to be framed in accordance with the spirit and genius of the time and country in which they are enacted, so it is perfectly just that they be modified by the genius and spirit of succeeding ages. If the conditions of our social relations be now very different from those which existed at the beginning of the sixteenth century, it may fairly be presumed that some changes will be requisite to adapt institutions then first called into existence, to the intellectual necessities of the present day. But there is an immeasurable distance between adaptation and subversion. And although it be utterly impossible for error to acquire any title to respect by reason of its antiquity, it is a safe precept to proceed very cautiously in changing what the collected wisdom of past ages may have established or sanctioned. Alteration and improvement are by no means synonymous: and it is so much easier to condemn than to reform, that it is a virtue of old institutions to be slow in their decisions, lest future experience should unhappily demonstrate that they had mistaken change for amendment. The College of Physicians has proceeded, and is proceeding, with a revision of its laws, which, it may be confidently predicted, will be productive of two important modifications: a restriction upon the admission of graduates of the English Universities to the Fellowship of the

College, and a greater facility for the election of licentiates; for it is the unanimous voice of the College, that every licentiate, educated as a physician, who may become honorably distinguished by professional eminence, or whose labours may have led to useful discoveries in medical science, should be invited to the Fellowship; but the College will never concede to a threatening adversary one single privilege which it is bound, as a national establishment, to withhold." (P. 111.)

